# Comparison of the Microhardness of Primary Dentin With Artificially Induced Caries Following the Application of Sodium Fluoride Varnish With the Intensive Technique Versus Fluoride Therapy With Silver Diamine Fluoride

**DOI:** 10.1155/ijod/7229454

**Published:** 2026-02-06

**Authors:** Sima Rafiei, Ahmad Jafari Ghavam Abad, Mehrsa Paryab, Golnaz Tayebi, Shima Younespour

**Affiliations:** ^1^ School of Dentistry, Tehran University of Medical Sciences, Tehran, Iran, tums.ac.ir; ^2^ Department of Pediatric Dentistry, School of Dentistry, Research Center for Caries Prevention, Dentistry Research Institute, Tehran University of Medical Sciences, Tehran, Iran, tums.ac.ir; ^3^ Department of Pediatric Dentistry, School of Dentistry, Tehran University of Medical Sciences, Tehran, Iran, tums.ac.ir; ^4^ Department of Dental Biomaterials, School of Dentistry, Tehran University of Medical Sciences, Tehran, Iran, tums.ac.ir; ^5^ Dental Research Center, Dentistry Research Institute, Tehran University of Medical Sciences, Tehran, Iran, tums.ac.ir

**Keywords:** deciduous, dentin, hardness, silver diamine fluoride, sodium fluoride, tooth

## Abstract

**Background and Objective:**

Black discoloration is a major challenge encountered when using silver diamine fluoride (SDF) to manage early childhood caries (ECC). This study aimed to assess the carious primary dentin microhardness following the application of sodium fluoride (NaF) varnish with the intensive technique versus fluoride therapy with SDF.

**Materials and Methods:**

In this in vitro study, 45 extracted relatively sound primary molars were randomly assigned to three groups: Group 1: Application of 30% SDF, Group 2: Application of Aria Dent varnish (5% NaF) three times within 10 days, and Group 3: Application of MI varnish (5% NaF + casein–phosphopeptide amorphous calcium phosphate [CPP‐ACP]) three times within 10 days. After sectioning and dentin surface preparation, dentin microhardness was measured at four different time points: baseline, after demineralization, and 24 h and 45 days after the application of the respective fluoride product. The teeth were under pH‐cycling throughout the entire study period. The microhardness values were compared among the groups, and at different time points using the generalized estimating equations (GEEs) model.

**Results:**

The mean microhardness at 24 h after treatment was not significantly different from that after demineralization in any group (*p* > 0.05). The mean microhardness significantly decreased in the 30% SDF group and significantly increased in the two NaF varnish groups at 45 days compared to 24 h (*p*  < 0.05). The pattern of change in microhardness was significantly different among the three groups (*p*  < 0.0001). MI varnish increased the microhardness significantly more than the other products within the 45‐day study period (*p*  < 0.05).

**Conclusions:**

The intensive protocol of NaF varnish application three times within 10 days, especially MI varnish that contains CPP‐ACP, may enhance the microhardness significantly more than SDF in the first month of use. This protocol may be able to serve as an efficient alternative to SDF.

## 1. Introduction

Early childhood caries (ECC) occurs due to factors such as nocturnal breast/bottle‐feeding, or nighttime bottle feeding with sugary drinks, and is still a major concern in children between 2 and 5 years [1]. Aside from night weaning and oral hygiene practice, application of remineralizing agents is also recommended to control or stop the progression of caries. Fluoride is the most commonly used remineralizing agent [2]. Many young children have carious lesions extended to dentin. The progression rate of dentin caries is much higher than that of enamel caries due to the different number and structure of hydroxyapatite crystals in dentin compared to enamel [3], and can lead to pulpal involvement, infection, and subsequent involvement of the underlying permanent tooth bud. Due to the young age of children and their dental fear and anxiety, advanced behavioral control methods such as sedation and general anesthesia may be required for definite treatment and final restoration of dentin caries in children, which are not often accepted by parents due to high cost and high risk of such procedures [4]. Therefore, preventing caries progression in young children is a priority for pedodontists.

Sodium fluoride (NaF) varnishes have long been prescribed for children under 6 years of age due to their safety and easy application. Their application is recommended every 3 months in children at high risk of caries [5]. Recently, silver diamine fluoride (SDF), which contains silver nitrate, gained popularity as an effective substance for caries cessation. It has been recommended by the American Academy of Pediatric Dentistry for caries control in children who cannot undergo general anesthesia for dental procedures for any reason. The efficacy of the abovementioned products for reinforcement of the tooth structure, and remineralization of carious enamel or dentin has been investigated and compared in the literature [6–14]. However, SDF application causes tooth discoloration due to penetration of silver ions into dentin, and formation of silver compounds; therefore, it is not well accepted by some parents [15]. Clinicians need to understand parental sensitivities regarding the staining effect of SDF in order to adequately plan for the use of SDF as a method of caries management in pediatric patients [16].

Application of 5% NaF varnish three times within 10 days is another recommended technique for management of acute caries [17], which has not received much attention. This study aimed to assess the effect of an intensive protocol of application of 5% NaF varnish three times within 10 days in comparison with 30% SDF on carious primary dentin microhardness during a 45‐day period. Given that optimal results are obtained, the intensive NaF varnish application protocol may be able to serve as an alternative to SDF without causing tooth discoloration. The null hypothesis of the study was that the application of NaF varnish with the intensive protocol would be as effective as fluoride therapy with SDF in improving the microhardness of primary carious dentin.

## 2. Materials and Methods

This in vitro, experimental study was ethically approved by the ethics committee of Tehran University of Medical Sciences (IR.TUMS.BLC.1402.109). The sample size was calculated to be 15 teeth in each group according to a study by Farhadian et al. [12], using one‐way ANOVA feature of Minitab Version 18 software, and assuming *α* = 0.05, *β* = 0.20, largest mean difference of 55 among the groups, similar standard deviation values of 46.80, and power of 0.80.

## 3. Methodology

A total of 45 primary molars extracted due to caries or for orthodontic reasons were used in this study. The inclusion criteria were presence of at least three buccal, lingual, and proximal walls with no caries or cracks, and presence of half or two‐thirds of the root. The teeth were disinfected with chloramine solution and rinsed with distilled water. The teeth were collected over a period of 3 months and stored in distilled water at 37°C temperature. Next, they were buccolingually sectioned by a diamond disc and high‐speed handpiece. The enamel was removed to obtain a sound dentin specimen free from caries while preserving the tooth anatomy. The specimens were then mounted in auto‐polymerizing acrylic resin (Acropars, Tehran, Iran), and the dentin surface was polished with 800‐, 1000‐ and 2000‐grit silicon carbide abrasive papers to smoothen the surface and maximize the microhardness measurement accuracy. Subsequently, the specimens were immersed in distilled water for 24 h to eliminate the smear layer caused by polishing.

After removal from distilled water, all specimens underwent microhardness testing in a Vickers hardness tester (V‐Test II 160; Baresiss, Germany) by application of 100 g load for 10 s to obtain the baseline microhardness (VHN0). The microhardness of each specimen was measured at three points with a minimum distance of 100 µm from each other, and the mean of the three values was calculated and reported as the final Vickers hardness number (VHN0). The specimens were then immersed in a demineralizing solution composed of 2.2 mM CaCl_2_, 2.2 mM NaH_2_PO_4_, and 0.05 M acetic acid with a pH of 4.4 adjusted by 1 M KOH for 10 h to simulate dentin caries by causing 60% hardness reduction. The 10‐h immersion time was selected according to a pilot study, and through comparison of the microhardness after different immersion times of 5, 8, 10, and 15 h.

The specimens were then dried and cleaned with cotton rolls, and their secondary microhardness (VHN1) was measured as explained earlier. The reduction in microhardness compared with the baseline value was calculated and reported. At this step, eight teeth were excluded from the study due to excessive microhardness reduction exceeding the normal range, and were replaced with eight other eligible specimens. This was done to exclude teeth with a different (compromised) dentin structure. The extreme decrease in microhardness in some specimens could be a sign of weak or brittle dentin in the tooth, which could cause data variability. Thus, we tried to ensure that the specimens tested were within the normal range of microhardness reduction.

Next, selected specimens were randomly assigned to three experimental groups (*n* = 15 per group) using a computer‐generated random number sequence (Microsoft Excel, random function). The allocation sequence was prepared by an investigator not involved in specimen preparation or microhardness testing to ensure allocation concealment and minimize selection bias.

Next, the specimens were stored in distilled water for 24 h. Fluoride application was then performed in the three groups as follows:

Group 1: The specimens were removed from distilled water after 24 h, and completely dried with air spray. Next, 30% SDF (Cariestop; Biodynamica Company, Brazil) was rubbed on the surface of the specimens in the form of a thin coat by a microbrush. After 3 min, the specimens were immersed in artificial saliva as the remineralizing solution with the composition of 1.5 mM CaCl_2_, 0.9 mM NaH_2_PO_4_, and 0.15 M KCl with an adjusted pH of 7.0. After 24 h, they were dried with a cotton roll, and their microhardness was measured for the third time (VHN2).
Group 2: The specimens were removed from distilled water and completely dried with air spray. Next, Aria Dent varnish containing 5% NaF (Asia‐ Chemi Teb company, Tehran, Iran) was applied on the surface of the specimens three times with equal intervals within 10 days. It was rubbed as a thin coat by using a microbrush and after 1 min, the specimens were immersed in artificial saliva. After 24 h, excess material was removed by a soft toothbrush, and the specimens were placed back in artificial saliva. After 4 and 9 days, NaF varnish was reapplied on the specimens as explained above, and they were stored in artificial saliva; 24 h after the third application, they were dried with a cotton roll, and their microhardness was measured for the third time (VHN2).
Group 3: The specimens in this group received MI varnish containing 5% NaF + casein–phosphopeptide amorphous calcium phosphate (CPP‐ACP; GC Corporation, Japan) three times within 10 days as explained for Group 2, and the microhardness of the specimens was then measured for the third time (VHN2).

After the measurement of VHN2, the specimens in all three groups underwent demineralization–remineralization cycles (pH‐cycling) simulating the oral environment for 45 days. For this purpose, they were immersed in a demineralizing solution with a pH of 4.5 for 3 h/day followed by immersion in artificial saliva as a remineralizing agent with a pH of 7.2 for 21 h [18]. The final microhardness (VHN3) was measured on day 45.

It should be noted that in all stages, the microhardness test was performed by an experienced operator who was blinded to the group allocation of the specimens.

All statistical analyses were conducted using SPSS Version 26.0 (SPSS Inc., Chicago, IL, USA), and the level of significance was set at *α* = 0.05. The Shapiro–Wilk test was used to assess the normality of data distribution.

Data were analyzed using the generalized estimating equations (GEEs) model to account for the correlation between repeated measures obtained from each tooth at different time points (VHN0 : baseline, VHN1: after demineralization, VHN2 : 24 h after fluoride application, and VHN3 : 45 days after fluoride application). The GEE model with an identity link function and exchangeable working correlation matrix was applied to evaluate changes in microhardness over time and to compare differences among the three treatment groups. Pairwise comparisons were adjusted using the Bonferroni correction. Partial *η* ^2^ values were computed from Wald chi‐square statistics (*η* ^2^ = *χ* ^2^ / [*χ* ^2^ + df]) to quantify the standardized effect size for each model effect.

## 4. Results

A total of 45 primary dentin specimens were evaluated and distributed equally among the three experimental groups.

Table 1 presents the descriptive statistics of dentin microhardness values at baseline (VHN0), after demineralization (VHN1), 24 h after fluoride application (VHN2), and after 45 days of pH‐cycling (VHN3).

**Table 1:** Descriptive statistics of microhardness at baseline, after demineralization, at 24 h, and at 45 days after the treatment in the three study groups.

Study groups	Measurement time			
	Baseline	After demineralization	24 h	45 days
Group 1				
Mean ± SD	61.16 ± 6.99	26.49 ± 8.47	25.94 ± 7.53	16.64 ± 5.70
Median	62.10	24.97	23.28	15.57
Minimum to maximum	45.87 to 71.70	14.57 to 40.63	17.83 to 44.53	11.13 to 34.93
Group 2				
Mean ± SD	66.17 ± 6.98	25.77 ± 7.33	26.62 ± 5.89	30.25 ± 5.98
Median	64.60	24.60	24.84	28.90
Minimum to maximum	57.30 to 79.62	14.13 to 39.77	19.90 to 39.25	21.80 to 41.87
Group 3				
Mean ± SD	60.98 ± 9.86	24.19 ± 7.33	23.60 ± 5.42	34.11 ± 4.75
Median	58.87	23.12	22.83	35.13
Minimum to maximum	49.63 to 83.70	13.60 to 39.83	16.20 to 35.30	24.63 to 41.67

*Note*: study groups: Group 1: 30% silver diamine fluoride; Group 2: 5% sodium fluoride Aria Dent varnish; Group 3: 5% sodium fluoride MI varnish.

Abbreviation: SD, standard deviation.

**Figure 1:**
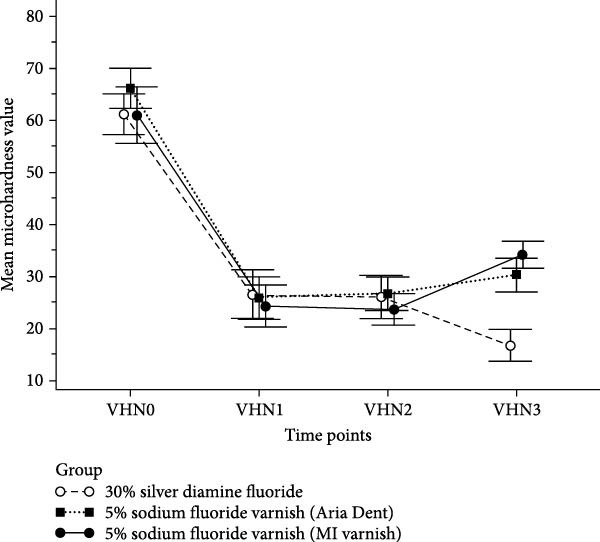
Changes in mean microhardness of primary dentin over time for the three treatment groups. Error bars represent 95% confidence intervals. Study time points: baseline (VHN0), after demineralization (VHN1), 24 h after fluoride application (VHN2), and 45 days after fluoride application (VHN3).

The GEE model revealed significant effects of group (Wald *χ* ^2^ = 7.04, df = 2, *p* = 0.03, partial *η* ^2^ = 0.78), time (Wald *χ* ^2^ = 661.84, df = 3, *p* < 0.001, and partial *η* ^2^ = 0.99), and a highly significant group × time interaction (Wald *χ* ^2^ = 132.51, df = 6, *p* < 0.001, and partial *η* ^2^ = 0.96). According to this analysis, the pattern of microhardness change over time differed among the three groups (Figure 1).

Within group comparisons demonstrated that in all groups, microhardness significantly decreased after demineralization compared with baseline (*p* ≤ 0.001; Table 2).

**Table 2:** Estimated pairwise differences in the mean microhardness between time points within each group based on generalized estimating equations model.

Pairwise difference of groups	Group 1		Group 2		Group 3	
	Mean difference (95% CI)	*p*‐Value	Mean difference (95% CI)	*p*‐Value	Mean difference (95% CI)	*p*‐Value
VHN1–VHN0	34.67 (−43.09 to ‐26.25)	*p* < 0.001	40.40 (−47.33 to ‐33.47)	*p* < 0.001	36.79 (−47.35 to −26.24)	*p* < 0.001
VHN2–VHN0	35.21 (−44.02 to −26.40)	*p* < 0.001	−39.56 (−47.19 to −31.92)	*p* < 0.001	37.38 (−46.76 to −28.00)	*p* < 0.001
VHN3–VHN0	44.51 (−52.67 to −36.36)	*p* < 0.001	35.92 (−43.99 to −27.85)	0.001	26.87 (−36.89 to −16.85)	*p* < 0.001
VHN2–VHN1	0.55 (−4.42 to 3.33)	1.00	0.84 (−3.05 to 4.75)	1.00	−0.58 (‐−4.06 to 2.89)	1.00
VHN3–VHN1	−9.84 (−15.77 to −3.92)	*p* < 0.0001	4.48 (−0.01 to 8.98)	0.052	9.92 (5.31 to 14.53)	*p* < 0.0001
VHN3–VHN2	9.30 (−13.91 to −4.69)	*p* < 0.0001	3.64 (0.19 to 7.08)	0.025	10.51 (6.78 to 14.23)	*p* < 0.001

*Note*: study groups: Group 1: 30% silver diamine fluoride; Group 2: 5% sodium fluoride Aria Dent varnish; Group 3: 5% sodium fluoride MI varnish. Study time points: VHN0: baseline, VHN1: after demineralization, VHN2: 24 h after the application of fluoride, VHN3: 45 days after the application of fluoride.

Abbreviation: CI, confidence interval.

At 24 h after treatment (VHN2), no significant change in microhardness was observed compared with the post‐demineralization stage (VHN1) in any of the groups (*p* = 1.00; Table 2). However, after 45 days (VHN3), the groups exhibited distinct trends. In the SDF group (Group 1), microhardness significantly decreased compared with the 24‐h measurement (mean difference = – 9.30; *p*  < 0.0001). In contrast, the Aria Dent NaF group (Group 2) showed a significant increase in microhardness after 45 days (mean difference = + 3.64; *p* = 0.025). The MI varnish group (Group 3) demonstrated the greatest increase in microhardness over the same period (mean difference = + 10.51; *p*  < 0.001).

The interaction effect of time and treatment group on dentin microhardness was analyzed using the GEE model, and pairwise comparisons of these interactions are presented in Table 3.

**Table 3:** Between group comparisons of microhardness changes over time based on time–group interaction using the generalized estimating equations model.

Group comparison	Group 2–Group 1		Group 3–Group 1		Group 3–Group 2	
	Mean difference (95% CI)	*p*‐Value	Mean difference (95% CI)	*p*‐Value	Mean difference (95% CI)	*p*‐Value
VHN1–VHN0	−5.73 (−12.08 to 0.61)	0.08	−2.12 (−9.98 to 5.73)	0.60	3.61 (−3.74 to 10.96)	0.34
VHN2–VHN0	4.34 (−11.13 to 2.44)	0.21	2.16 (−9.66 to 5.33)	0.57	2.18 (−4.86 to 9.22)	0.54
VHN3–VHN0	8.59 (1.91 to 15.27)	0.01	17.64 (10.12 to 25.16)	*p* < 0.0001	9.05 (1.56 to 16.54)	0.02
VHN2–VHN1	1.39 (−1.81 to 4.59)	0.39	0.04 (−3.07 to 2.99)	0.98	1.43 (−4.47 to 1.61)	0.36
VHN3–VHN1	14.32 (10.00 to 18.65)	*p* < 0.0001	19.77 (15.40 to 24.13)	*p* < 0.001	5.44 (1.69 to 9.19)	0.004
VHN3–VHN2	12.93 (9.58 to 16.28)	*p* < 0.0001	19.80 (16.35 to 23.26)	*p* < 0.001	6.87 (3.92 to 9.83)	*p* < 0.0001

*Note*: *p*‐Values are reported using Bonferroni adjustment. Study Groups: Group 1: 30% silver diamine fluoride; Group 2: 5% sodium fluoride Aria Dent varnish; Group 3: 5% sodium fluoride MI varnish. Study time points: VHN0: baseline, VHN1: after demineralization, VHN2: 24 h after the application of fluoride, VHN3: 45 days after the application of fluoride.

Abbreviation: CI, confidence interval.

No statistically significant differences were found in the pattern of microhardness change among the groups during the early stages (VHN1–VHN0 and VHN2–VHN0; *p* > 0.05). However, from the demineralized stage to 45 days (VHN3–VHN1), both NaF varnish groups showed significantly greater recovery in microhardness compared with SDF (mean difference = 14.32; *p* < 0.0001 for Aria Dent and 19.77; *p* < 0.001 for MI varnish). The MI varnish group also showed a significantly greater increase than Aria Dent group (mean difference = 5.44; *p* = 0.004).

Similarly, for the period between 24 h and 45 days (VHN3–VHN2), the NaF groups again demonstrated higher gains compared with SDF (mean difference = 12.93; *p*  < 0:0001 for Aria Dent and 19.80; *p*  < 0.001 for MI varnish), with MI varnish outperforming Aria Dent (mean difference = 6.87; *p*  < 0.0001).

## 5. Discussion

Mechanical caries removal and cavity restoration are the ideal goals in treatment of ECC. However, in case of poor patient cooperation, and when sedation and general anesthesia are not an option due to high cost or the associated risks, cessation of caries progression by the application of remineralizing agents such as fluoride may serve as a noninvasive and low‐cost alternative [19].

The hard tooth structure is composed of enamel and dentin. Microscopic assessments have shown that hydroxyapatite crystals are larger in dentin and have a higher carbonate content compared with the enamel. Resultantly, dentin is less resistant to acidic dissolution and is remineralized slower than the enamel [3]. This difference is more significant in primary dentin due to its lower mineral content than permanent dentin [6]. Therefore, much more efficient remineralizing agents are required to stop caries progression in dentin.

SDF contains 44,800 ppm fluoride, and was first introduced and approved as a desensitizing agent. It later gained attention for caries cessation. In 2018, the American Dental Association recommended annual application of 38% SDF for cessation of advanced cavitated carious lesions in coronal surfaces of primary teeth according to systematic reviews and meta‐analyses [5]. The caries cessation mechanism of SDF is based on its high fluoride content, and presence of silver in its composition, which has antibacterial activity and creates a silver chloride protective layer on the surface [20]. Aside from the positive cariostatic efficacy of silver, it causes tooth discoloration, which is a clinical concern. Therefore, it is highly important for dental clinicians to stop dentin caries by the application of remineralizing agents that do not cause black discoloration. In vitro and clinical studies have demonstrated that application of NaF varnish three times within 10 days maximizes its efficacy [21–24].

Considering the significance of management of ECC, this study assessed the efficacy of an intensive protocol of NaF varnish application three times within 10 days compared with SDF for enhancement of primary dentin microhardness. Accordingly, Aria Dent varnish (5% NaF) and MI varnish (5% NaF + CPP‐ACP) were used, and microhardness testing was performed for assessment of their efficacy in the current study. Microhardness testing enables reproducible measurement of microhardness of the same specimen at different time points, and has optimal accuracy and reliability [25].

Some previous studies compared the efficacy of NaF varnishes with and without CPP‐ACP and SDF. The majority of such studies evaluated the enamel of primary teeth [6, 9] and permanent teeth [13, 14] and indicated the greater or comparable efficacy of NaF varnish, compared with SDF. Some others reported higher efficacy of SDF for remineralization of demineralized enamel [8, 10, 11]. Another study showed that the positive efficacy of SDF for caries cessation was related to the simultaneous application of NaF varnish [26].

Studies on dentin structure are fewer in number. Yýlmaz et al. [7], in 2020 evaluated the remineralizing effects of 38% SDF and 5% NaF on artificially induced caries in primary dentin and its mineral density. They showed an increase in mineral density in both groups; however, the primary dentin mineral density was significantly greater in the SDF group than the NaF group. It appears that SDF is more effective on dentin than NaF varnishes. Due to the large molecular size of silver, it has high affinity to inhibit dentin collagenase and stop collagen degradation; whereas, tooth enamel has a lower protein, carbonate, and phosphate content. On the other hand, NaF varnish has a small amount of free fluoride ions, which are released following saliva penetration into the varnish matrix. Therefore, it needs long‐term exposure to the saliva. In addition to exposure time, differences in the matrix and the mechanism of combination or dissolution of NaF in it play important roles in optimal efficacy of the varnish [2]. Considering the mechanism of action of varnishes, it appears that their repeated application within a short period of time would yield the expected positive results for caries control.

The present results revealed that application of remineralizing agents did not cause a significant change in microhardness after 24 h. However, after 45 days of pH‐cycling, the positive effect of NaF varnishes (applied three times within 10 days) on microhardness was greater than that of SDF and they slightly, but significantly, increased the dentin microhardness. These results were different from the findings of Yýlmaz et al. [7], which may be due to the fact that NaF varnish was used intensively in the present study (three times within 10 days). Farhadian et al. [12] and Sivapriya et al. [27] used fluoride varnish with a higher application frequency in a short period of time, and noticed its improved efficacy for remineralization of demineralized permanent enamel. It appears that using the intensive fluoride protocol is safe as the time interval is 3–5 days, which is adequate for excretion by the kidneys, and there is no safety concern associated with this technique.

Also, it should be noted that 30% SDF was used in the present study. Although the American Academy of Pediatric Dentistry recommends the application of 38% SDF for caries control, application of 30% SDF for 3 min has been reported to be equally effective [28]. Therefore, considering the existing concerns regarding fluorosis [29], 30% SDF, which is commonly available and used by pedodontists, was applied in the current study. Nonetheless, it may be considered as a limitation of this study, and future studies are recommended to investigate and compare the use of 38% SDF and the subsequent application of potassium iodide. Another methodological difference between the present study and that of Yýlmaz et al. [7] was that dentin microhardness was evaluated over a longer period of time in the current study. Surendranath et al. [30] reported that the positive efficacy of SDF application for microhardness enhancement was only observed for less than 1 month, and only its antibacterial effects remained for a longer period of time.

Pairwise comparisons of the study groups in the present study revealed that the efficacy of 5% NaF MI Varnish for microhardness enhancement was significantly higher than that of 5% NaF Aria Dent varnish. Similarly, another study assessed the efficacy of fluoride combined with CPP‐ACP compared with NaF varnish, and reported superior remineralization of primary teeth following addition of tricalcium phosphate to NaF varnish [31]. This formula was found to be the best as it releases the maximum amount of fluoride in ppm in artificial saliva for up to 3 months [32]. Also, some studies have shown that CPP‐ACPF varnish appears to be equally effective as other fluoride varnishes in remineralizing artificially induced white spot lesions [6, 33]. In the present study, pH‐cycling was performed for a longer period, 45 days instead of 24 h and 1 week, to assess the substantivity of the effect of fluoride products on dentin. The solutions and the artificial saliva were refreshed daily in order to prevent reduction in efficacy by the materials released from dentin. The fluoride present in pH‐cycling solutions simulates the role of fluoride present in the human saliva or fluoride received from foods and drinks. Nonetheless, it should be noted that many influential factors such as oral microorganisms, variations in the saliva composition, and oral hygiene techniques cannot be simulated in vitro. It appears that the synergistic effect of silver and fluoride would increase the success of SDF in the clinical setting compared with in vitro. Chhattani et al. [34] demonstrated that SDF caused a significant reduction in cariogenic bacterial count compared with NaF varnish. Thus, clinical studies are recommended to assess the efficacy of the intensive protocol in children with ECC and also its parental acceptance.

## 6. Conclusion

The results revealed that the effect of one‐time application of SDF on microhardness decreased with time, and the intensive protocol of NaF varnish application three times within 10 days, especially when using MI Varnish containing CPP‐ACP and higher levels of calcium and phosphorus, may yield more favorable results in the first month of ECC management when a greater effect is expected from the applied products. However, it should be noted that parental acceptance must be considered.

## Disclosure

The Research Center for Caries Prevention, Dentistry Research Institute, Tehran University of Medical Sciences, is a non‐profit research center, and the provided grant did not influence either the method used for data collection, or the data analysis and results. This study was derived from a thesis for a DDS degree.

## Conflicts of Interest

The authors declare no conflicts of interest.

## Funding

This work was supported by a research grant from the Research Center for Caries Prevention, Dentistry Research Institute, Tehran University of Medical Sciences (Grant 1402‐3‐234‐68833).

## Data Availability

This article is the results of an experimental research done on tooth samples. All data obtained in the laboratory are available.
